# *MYB *suppresses differentiation and apoptosis of human breast cancer cells

**DOI:** 10.1186/bcr2614

**Published:** 2010-07-26

**Authors:** Yvette Drabsch, Ramsay G Robert, Thomas J Gonda

**Affiliations:** 1 University of Queensland Diamantina Institute, University of Queensland, Ipswich Road, Brisbane, Queensland, 4102, Australia; 2 Peter MacCallum Cancer Centre, East Melbourne and the Pathology Department, The University of Melbourne, St Andrews Place, Parkville, Victoria, 3002, Australia

## Abstract

**Introduction:**

*MYB *is highly expressed in estrogen receptor positive (ER + ve) breast tumours and tumour cell lines. We recently demonstrated that *MYB *is essential for the proliferation of ER + ve breast cancer cells, and have now investigated its role in mammary epithelial differentiation.

**Methods:**

MCF-7 breast cancer cells were treated with sodium butyrate, vitamin E succinate or 12-O-tetradecanoylphorbol-13-acetate to induce differentiation as measured by Nile Red staining of lipid droplets and β-casein expression. The non-tumorigenic murine mammary epithelial cell (MEC) line, HC11, was induced to differentiate with lactogenic hormones. *MYB *levels were manipulated by inducible lentiviral shRNA-mediated knockdown and retroviral overexpression.

**Results:**

We found that *MYB *expression decreases following chemically-induced differentiation of the human breast cancer cell line MCF-7, and hormonally-induced differentiation of a non-tumorigenic murine mammary epithelial cell (MEC) line, HC11. We also found that shRNA-mediated *MYB *knockdown initiated differentiation of breast cancer cells, and greatly sensitised them to the differentiative and pro-apoptotic effects of differentiation-inducing agents (DIAs). Sensitisation to the pro-apoptotic effects DIAs is mediated by decreased expression of *BCL2*, which we show here is a direct *MYB *target in breast cancer cells. Conversely, enforced expression of *MYB *resulted in the cells remaining in an undifferentiated state, with concomitant suppression of apoptosis, in the presence of DIAs.

**Conclusions:**

Taken together, these data imply that *MYB *function is critical in regulating the balance between proliferation, differentiation, and apoptosis in MECs. Moreover, our findings suggest *MYB *may be a viable therapeutic target in breast cancer and suggest specific approaches for exploiting this possibility.

## Introduction

There is a critical balance between proliferation, differentiation, and apoptosis in the cellular composition of every tissue. In the hematopoietic system, *MYB *clearly plays a role in maintaining this balance. *MYB *is essential for hematopoiesis [1], is highly expressed in immature hematopoietic cells and its expression is down-regulated upon differentiation [2,3]. Moreover, enforced expression of normal and activated forms of MYB can suppress differentiation and maintain proliferation of hematopoietic cells [4-6].

For these reasons, most work on *MYB *has focused on its role in normal and leukemic hematopoiesis. However, there is increasing evidence for a role of *MYB *in colonic epithelial cell differentiation and homeostasis, and notably, in colon cancer [7]. The pattern of *MYB *expression in normal colonic crypts suggests that, similarly to the hematopoietic system, expression is high in immature, rapidly proliferating cells, and decreases with differentiation and maturation [8,9], whereas reduced *MYB *activity perturbs colonic epithelial proliferation, differentiation and viability [8,9]. The involvement of *MYB *in epithelial tumors was first suggested by the amplification of *MYB *in certain colon carcinoma-derived cell lines [10], and by its expression in a substantial proportion of tumors [7,11]. Moreover *MYB *expression in colon tumors correlates with poor clinical prognosis [12], and an important transcriptional regulatory region of *MYB *is frequently mutated in this disease [13,14]. Furthermore, *MYB *is required for colon carcinoma cell proliferation [7,15], and is down-regulated during differentiation of these cells [9] while, conversely, ectopic *MYB *expression can suppress their differentiation [16].

By contrast, much less is known about the functions of *MYB *in mammary epithelial cells. Nevertheless, it has been shown that *MYB *is expressed at relatively high levels in estrogen receptor (ER) positive breast cancers and tumor cell lines [17]. Moreover we, and others [18] have previously shown that *MYB *is a direct target of estrogen/ER signaling, and that *MYB *expression in breast cancer cells is regulated by transcriptional attenuation within its first intron [19]. Importantly, we have also recently shown that *MYB *is required for the proliferation of ER positive, but not ER negative, breast cancer cell lines [19], identifying for the first time a functional role for *MYB *in breast cancer. In addition, Fang *et al *[20] reported a prolactin-inducible association between MYB and Stat5a, and that a number of Stat5a-responsive promoters such as that of the *CISH *gene are further stimulated by MYB. Their results suggested that MYB may act as a coactivator for Stat5a, and also supported a proliferative function for *MYB *in human breast cancer.

To further understand the function of *MYB *in breast cancer and in mammary epithelial cells (MECs) generally, we have now investigated its role in the differentiation of these cells. Differentiation of human breast cancer cell lines (MCF-7 and ZR-75-1) and non-tumorigenic MEC (HC11) can be induced by chemical agents or lactogenic hormones, respectively, and results in morphological and molecular properties that are characteristic of mature ductal epithelial cells. We have found that mammary carcinoma cell lines in which *MYB *expression was 'knocked-down' by shRNA show changes that indicate differentiation has occurred in some of these cells. Moreover, these *MYB *knock-down cells are more sensitive to differentiation after exposure to low doses of differentiation-inducing agents, and can be driven into apoptosis with doses that would normally only induce differentiation. Conversely, ectopic expression of *MYB *suppressed differentiation and apoptosis induced by differentiation-inducing agents (DIAs). Taken together, our data show that *MYB *plays an important role in regulating the balance between proliferation, differentiation, and apoptosis in both normal and malignant mammary epithelial cells, and that this role is remarkably similar to that it plays in hematopoietic and colonic epithelial cells. Finally, our observation that DIAs and *MYB *inhibition synergize in killing breast tumor cells suggests an approach to developing new treatments for ER/MYB positive breast cancer.

## Materials and methods

### Cell culture

The breast cancer cell lines MCF-7 and ZR-75-1 were cultured in DMEM (Invitrogen, Mount Waverley, Vic, Australia) supplemented with 10% FBS, l-glutamine, penicillin G, and streptomycin sulfate (All from GIBCO/BRL, Grand Island, New York, USA). All cell lines were maintained at 37°C in a humidified 5% carbon dioxide/95% air incubator. Prior to reaching confluence, cells were trypsinized with a 0.05% trypsin/0.53 mM EDTA solution and resuspended in fresh growth medium before plating onto a new growth surface.

Sodium butyrate, vitamin E succinate, and 12-O-tetradecanoylphorbol-13-acetate were purchased from Invitrogen (Mount Waverley, Vic, Australia).

HC11 cells were grown in Roswell Park Memorial Institute medium (RPMI)-1640 (Invitrogen, Mount Waverley, Vic, Australia) medium containing 10% FBS, 5 μg/ml insulin, and 10 ng/ml epidermal growth factor (both from Sigma-Aldrich, Castle Hill, NSW, Australia). Differentiation was induced three days after reaching confluence in medium containing 5 μg/ml insulin, 1 μg/ml hydrocortisone and 5 μg/ml prolactin (Sigma-Aldrich, Castle Hill, NSW, Australia).

For lipid droplet staining, cells grown on glass cover slips were rinsed twice with PBS and fixed for 20 minutes with PBS containing 4% paraformaldehyde at 20°C. After another PBS rinse and staining for 15 minutes with Nile Red and 4',6-diamidino-2-phenylindole (DAPI) (Sigma-Aldrich, Castle Hill, NSW, Australia), the cells were PBS washed and mounted. Fluorescence imaging was performed by using automated excitation and emission filter wheels of a Fluorescent AxioSkop 2 plus Microscope (Carl Zeiss Pty Ltd, New South Wales, Australia). For flow cytometric analysis, cells not grown on glass cover slips were washed in PBS, and resuspended in PBS containing 0.01% Nile Red for 15 minutes at 37°C. After three washes in PBS, they were analyzed by flow cytometry using a FACS Calibur instrument (Becton Dickinson, San Jose, CA, USA), and the primary data were then processed using CellQuest software (Becton Dickinson, San Jose, CA, USA).

For siRNA transfection experiments, MCF-7 cells were plated and transfected the following day with 100 nM of BCL2 specific, or and control siRNAs (Dharmacon Research, Lafayette, CO, USA) by using lipofectamine 2000 (Invitrogen, Mount Waverley, Vic, Australia). Briefly, all transfections were performed in a mixture of Opti-MEM and complete media without antibiotics, as described previously [21]. The transfection incubation time for siRNA/lipofectamine 2,000 complexes was 24 hours.

### Chromatin immunoprecipitation assay

MCF-7 cells were grown to 95% confluence in phenol-red-free DMEM supplemented with 10% charcoal-stripped FBS for 72 hours; at which time, 10 nM b-estradiol was added for 12 hours. Chromatin immunoprecipitation (ChIP) was performed as previously described [22] by using rabbit immunoglobulin (Ig) G (Sigma, Castle Hill, NSW, Australia) and 1.1 anti-Myb/5.1 anti-Myb or 1.1 anti-Myb/Thelma anti-Myb monoclonal antibodies [23]. The resultant samples were used as real-time PCR templates to quantify binding of MYB to the various regions of *BCL2, MYC *or *GAPDH*. Primer sequences used in the PCR were: Myb binding sites (*MBS*) 1 F 5'-GCTCAGAGGAGGGCTCTTTC-3', MBS 1 R 5'-TTTCTCCTCCTCCTGGTCCT-3', *MBS *2 F 5'-CCCGCCTCTTCACCTTTCAG-3', *MBS *2 R 5'-CAATGGCACTTCAAGTCCCGA, *MBS *3 F-5'-GGTCAGGTGGACCACAGGT- 3', *MBS *3 R 5'-GTCCAAGAATGCAAAGCACA-3', *MBS *4 F 5'-CACAGCGCCAACAGAACTAC-3', *MBS *4 R 5'-ACAGGCCAGATGCCAGATAC-3', *MYC *F 5'-GCCTGCGATGATTTATACTCACAG-3', *MYC *R 5'-CGGAGATTAGCGAGAGAGGATC-3', *GAPDH *F 5'-ATCAATGGAAATCCCATCACCATCT-3', *GAPDH *R 5'-GGTTTTTCTAGACGGCAGGTCAG-3', Upstream Control F 5'-GCAGGTGCTCAACAGATGAA-3', Upstream Control R 5'-GGGATTGCCTTACAGGTGAA-3'.

### TUNEL

Apoptosis-induced nuclear DNA fragmentation was detected using the TMR Red *In Situ *Cell Death Detection Kit (Roche Diagnostics Corp, Indianapolis, IN, USA) following the manufacturer's protocol. Briefly, 24 hours after DIA treatment, cells grown on glass cover slips were fixed. This was followed by incubation in terminal deoxynucleotidyl transferase (TdT)-mediated dUTP nick end labeling (TUNEL) reaction mix for 60 minutes at 37°C. Slides were washed three times in PBS/Triton X-100/BSA (0.3%) and visualized on a fluorescent microscope. Cells not grown on glass cover slips were washed in PBS, and resuspended in PBS and TUNEL reaction mix for 60 minutes at 37°C. Cells were washed three times in PBS, and analysed by flow cytometry on the FACS Calibur as described earlier.

### Quantitative RT-PCR

Total RNA was prepared by using a RNeasy MiniKit (Qiagen, Valencia, CA, USA) according to the manufacturer's instructions. One microgram of total RNA was reverse transcribed in a total of 20 μl by using SuperScript III (Invitrogen, Carlsbad, CA, USA). The resulting cDNA was then diluted to a total volume of 100 μl with sterile water. Each real-time PCR consisted of 1 μl of diluted reverse transcriptase product, iQ SYBR Green Supermix (Bio-Rad, Hercules, CA, USA), and 50 nM forward and reverse primers (see below). Reactions were carried out on a RotorGene 3000 (Corbett Research, Sydney, NSW, Australia) at 95°C for 10 minutes, followed by 40 cycles of 95°C for 20 seconds, 56°C for 15 seconds, and 72°C for 20 seconds. Fluorescence measurements analyzed by using the RotorGene 3000 software. The fold-change expression of each gene was calculated by using the ΔΔCT method, with cyclophilin A as an internal control. Primers used for real-time PCR were: *MYB *F, 5'-GCCAATTATCTCCCGAATCGA-3'; *MYB *R, 5'-ACCAACGTTTCGGACCGT A-3'; *ß-casein *F, 5'-CCCTCAAATCCCAAAACTCA-3'; *ß-casein *R, 5'-GAGCAGAAGGGCTTGAACAG-3'; *BCL2 *F, 5'-GTTCGGTGGGGTCATGTGTGTGGAGAGCG-3'; *BCL2 *R 5'-TAGCTGATTCGACGTTTTGCCTGA-3'; *Cyclophilin *A F, 5'-GGCAAATGCTGGACCCAACACAAA-3'; *Cyclophilin *A R, 5'-CTAGGCATGGGAGGGAACAAGGAA-3'.

### Western blotting

Western blot analysis was conducted as described previously [24]. Briefly, extracts prepared in SDS loading buffer were resolved in SDS/10% PAGE gels and transferred to PVDF membranes. These were incubated overnight in the presence of anti-c-Myb antibody 1.1 [25] and were developed by using ECL western blotting substrates (Pierce Biotechnology, Rockford, IL, USA).

## Results

### Characterization of, and *MYB *expression during, the differentiation of human mammary carcinoma cell lines

**Figure 1:**
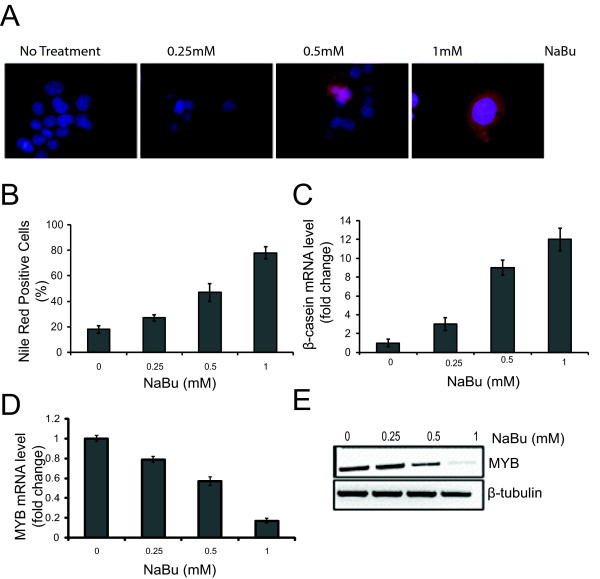
**Differentiation induction and cells treated with the differentiation-inducing agent NaBu**. **(a) **MCF-7 cells were grown on glass cover slips and treated with increasing amounts of sodium butyrate (NaBu), as indicated, for 72 hours. Morphological changes were evaluated by fluorescent microscopy (×63). Induction of biochemical differentiation was determined by using Nile Red stain for lipid vesicles in cells (red). Nuclear DNA was stained with 4',6-diamidino-2-phenylindole (DAPI) (blue). **(b) **Quantitative analysis of lipid induction. MCF-7 cells, as above, were stained with Nile Red and analyzed by flow cytometry as described in the Materials and methods. Standard deviations are shown as error bars (*n *= 6). **(c) **Quantitative (Q) PCR of ß-casein mRNA expression following treatment with increasing doses of NaBu. The induction of ß-casein is normalized against that seen in untreated cells. **(d) **Q-PCR of *MYB *mRNA from MCF-7. MCF-7 cells treated with the increasing dosage of NaBu show a dose dependent decrease in expression of *MYB *mRNA. Data from (c) and (d) represent mean values (*n *= 6), and standard deviations are shown as error bars. All Q-PCR data were normalized against *cyclophilin A *controls. **(e) **Western blot analysis of MYB from total cell lysates as above. A ß-tubulin loading control is also shown. Results are indicative of two independent experiments.

We first characterized the differentiation of MCF-7 (and ZR-75-1; data not shown) cells using several reported markers of this process. A number of compounds are capable inducing differentiation in breast cancer cells, such as sodium butyrate (NaBu), vitamin E succinate (VES) or 12-O-tetradecanoylphorbol-13-acetate (TPA) [26,27]. We examined the formation of lipid droplets as an indicator of breast cancer cell differentiation [28] using the fluorescent dye Nile Red in combination with fluorescence microscopy or flow cytometry [29]. Vehicle-only treated MCF-7 cells expressed only minimal numbers of detectable lipid vacuoles when stained with Nile Red (Figures 1a and 1b). By contrast, there was a dose-dependent increase in the accumulation of lipid droplets in the cytoplasm of the cells treated with NaBu for 72 hours, and in cells treated with VES and TPA [see Additional File 1].

As mammary epithelial cells undergo differentiation there is an increase in the transcription of the gene encoding the milk protein ß-casein [26]; as such, this can be used as a molecular marker for differentiation. Indeed, quantitative RT-PCR showed that ß-casein message was significantly increased after treatment of MCF-7 cells with increasing concentrations of DIAs for 72 hours (Figure 1c).

Previous studies have shown that differentiation of a number of different cell types, notably hematopoietic [2,3] and colonic epithelial cells [8,9,11,14,16], is associated with a reduction in *MYB *expression. Therefore, we asked whether the level of *MYB *would also be reduced when mammary carcinoma cells underwent differentiation. The *MYB *message was, as expected, readily detectable in control MCF-7 cells by quantitative RT-PCR, but treatment with increasing concentrations of NaBu for 72 hours induced a dose-dependent decrease in the level of *MYB *message (Figure 1d). Western blot analysis of total cell lysates showed a corresponding profile for the MYB protein (Figure 1e).

### *MYB *expression decreases with differentiation of non-tumorigenic mammary epithelial cells

**Figure 2:**
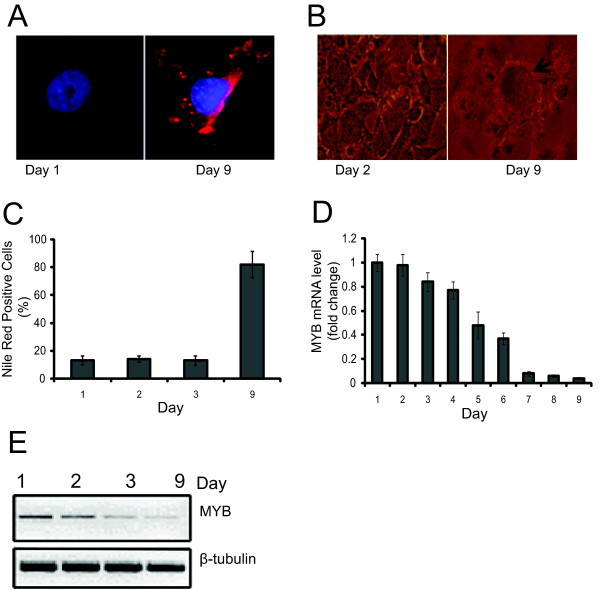
**Myb expression during confluence/hormone-induced differentiation of HC11 cells**. HC11 were proliferating (Day 1), grown to confluence (Day 2), and then differentiated by removing epidermal growth factor (EGF) for 24 hours (Day 3), followed by addition of differentiation medium containing lactogenic hormones (Days 4 to 9). **(a) **HC11 cells grown on glass cover slips were stained for DNA (4',6-diamidino-2-phenylindole [DAPI]; blue), lipid vesicles (Nile Red; red), and viewed under fluorescence microscopy at Day 1 and Day 9. **(b) **HC11 cells were grown in 24-well dishes, and induced to differentiate as described. Shown are light micrographs of confluent cells and 'domes' (arrow) which form in the presence of differentiation inducing medium. **(c) **Quantitative analysis of Nile Red staining by flow cytometry. HC11 cells were analyzed for lipid vesicle formation during the different stages of differentiation. **(d) **Quantitative PCR of *MYB *mRNA from HC11 cells undergoing differentiation. HC11 cells were analyzed each day during the differentiation process. Standard deviation is represented by error bars. **(e) **Western blot analysis of MYB protein from total cell lysates of HC11 cells during the differentiation process. A ß-tubulin loading control is also shown. Results are indicative of two independent experiments.

HC11 is an immortalized line of non-transformed mammary epithelial cells, which originated from mid-pregnant mouse mammary gland tissue, and has retained important characteristics of normal mammary epithelial cells [30]. Following stimulation with lactogenic hormones, these cells differentiate, synthesize ß-casein, and form blister-like structures, called 'domes' that are believed to result from fluid secretions. We first confirmed that HC11 MECs could undergo differentiation in the presence of lactogenic hormones, as monitored by the presence of lipid droplets detected by Nile Red (Figures 2a and 2c), the formation of domes (Figure 2b), and the induction of ß-casein expression (data not shown). In the course of such experiments, cell differentiation occurred progressively after removal of epidermal growth factor (EGF) for 24 hours and the subsequent addition of lactogenic hormones over seven days. Quantitative RT-PCR and western blot analysis demonstrated a decrease of the steady state levels of *MYB *mRNA and protein, respectively, as these cells underwent differentiation (Figures 2d and 2e). These data suggest that the reduction in *MYB *levels is part of the normal pathway of differentiation of mammary epithelial cells, and validate the use of mammary carcinoma cell lines and MECs as models to study the role of *MYB *in this process.

### *MYB *knockdown promotes differentiation of mammary carcinoma cells

We have previously used a doxycycline (Dox)-inducible lentiviral siRNA system targeting *MYB *to show that *MYB *is required for the proliferation of ER positive cell lines [19]. To examine the effect of *MYB *knockdown on differentiation, MCF-7 cells stably transduced with this vector, or appropriate controls, were treated with Dox for 72 hours. The cells were then stained for lipid droplet accumulation using Nile Red. Approximately 35% of *MYB *knockdown cells stained positively for lipid droplet accumulation in contrast to approximately 20% of control cells [see Additional file 2]. This is a modest, yet statistically significant (Student's t test, *n *= 6, *P *< 0.05), increase. It should be noted that the intensity of Nile Red staining was greater in the *MYB *knockdown cells, and the number of droplets/cell and droplet size in the *MYB *knockdown cells were greater than those seen in the control cells as visualized by fluorescence microscopy [see Figure S2A in Additional file 2]. In addition, ß-casein mRNA levels rose by approximately 3 fold following *MYB *knockdown [see Figure S2C in Additional file 2]. These experiments were repeated using ZR-75-1 cells, and a similar result was observed [see Additional file 3]. These data suggest that *MYB *knockdown induces breast tumor cells to initiate the process of differentiation in the absence of DIAs, albeit with limited efficiency.

### Synergy between DIA treatment and *MYB *knockdown

**Figure 3:**
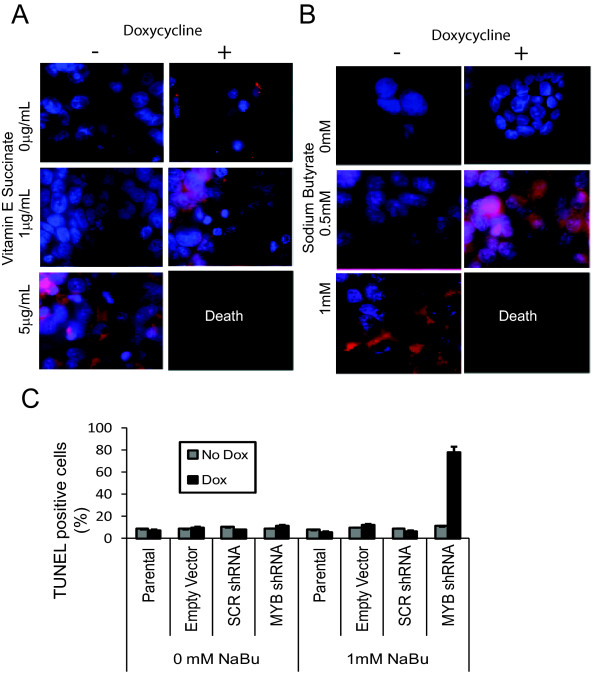
***MYB *knockdown sensitizes MCF-7 to differentiation-inducing agents**. **(a) **Vitamin E succinate (VES) was used to treat MCF-7 cells with a doxycycline (Dox)-inducible shRNA targeting *MYB *[19] MCF-7 cells grown on a glass coverslip, and treated with or without 5 mg/mL Dox for 24 hours before exposure to VES for three days, were then stained with Nile Red (red) for lipid vesicles and 4',6-diamidino-2-phenylindole (DAPI) (blue) for DNA detection. **(b) **Sodium butyrate (NaBu) was used to treat MCF-7 cells with the Dox-inducible shRNA targeted against *MYB*, as in (a). Results are indicative of at least two independent experiments. **(c) **Flow cytometric analysis of TUNEL staining for apoptotic cells. MCF-7 cells stably transfected with inducible shRNA treated for 24 hours with or without Dox, were grown in the presence or absence of 1 mM NaBu for a further 24 hours, and were then assayed for apoptosis using TUNEL. Standard deviation is represented by error bars.

As *MYB *knockdown in mammary carcinoma cells resulted in only a limited amount of differentiation, we next asked whether *MYB *knockdown was able to act in a synergistic manner with DIAs. *MYB *knockdown MCF-7 cells, and appropriate controls, were treated with or without Dox for 24 hours, and then treated with NaBu or VES for a further 72 hours. Staining for lipid droplet accumulation treated with or without Dox (Figures 3a and 3b) showed that the effect of *MYB *knockdown coupled with the lowest concentration of VES or NaBu caused differentiation comparable with that seen only with the highest concentration of DIAs used alone (Figure 1a). Strikingly, when *MYB *knockdown cells were treated with the higher concentration of the DIA, complete cell death, rather than differentiation, was observed (Figures 3a and 3b). Similarly-treated cells were then assayed by TUNEL after 24 hours of exposure to DIA. A high percentage of these cells appeared apoptotic on microscopic inspection, and FACS analysis of these cells showed that approximately 80% of had undergone apoptosis (Figure 3c); in contrast all control treatments resulted in a background level of 10 to 20% of TUNEL staining. Similar results were obtained using MCF-7 cells treated with TPA, and with ZR-75-1 cells treated with all three DIAs [see Additional file 4] (data not shown). Thus these data show that *MYB *knockdown sensitizes mammary carcinoma cells to differentiation and, at high concentrations of DIA, to apoptotic death.

### *BCL2 *is regulated by *MYB *in breast cancer cells

**Figure 4:**
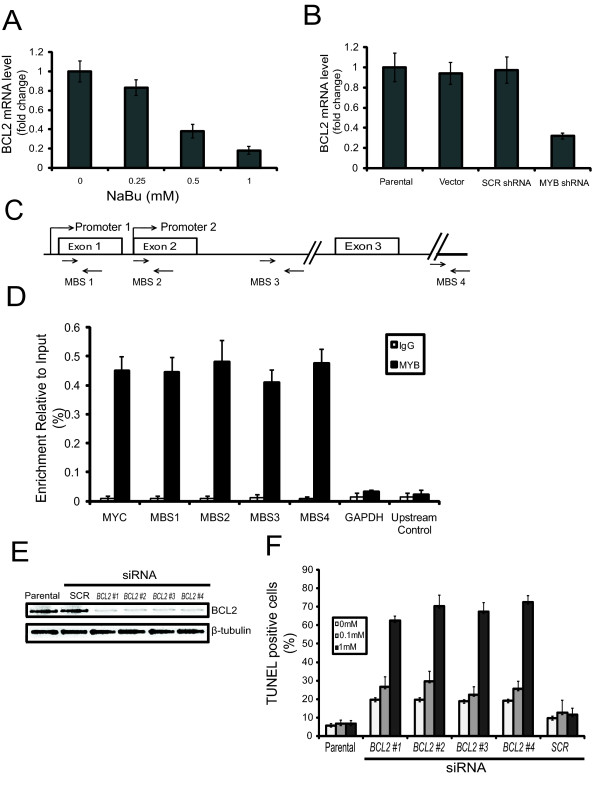
**Expression, *MYB *regulation and function of *BCL2 *in MCF-7 cells**. **(a) ***BCL2 *expression in MCF-7 cells treated with increasing doses sodium butyrate (NaBu). After 72 hours of treatment, cells were harvested and the mRNA was analyzed for expression of *BCL2 *by quantitative (Q) PCR. **(b) **MCF-7 cells stably transduced with an inducible *MYB *shRNA vector were grown in the presence of doxycycline (Dox) for three days, after which *BCL2 *was analyzed by Q-PCR. Standard deviation is represented by error bars. **(c) **A schematic of the *BCL2 *gene (not to scale) showing the relative locations of the primers (MBS-1-4) used for ChIP assays. **(d) **Q-PCR of MCF-7 cells assessed by ChIP assays using anti-MYB Ab. Rabbit IgG was used as a negative control. MYC is used as a positive control, and GAPDH and an upstream primer set are used as negative controls. **(e) **Western blot analysis of BCL2 knockdown by four siRNAs (#1-4), parental cells, and control siRNA. MCF-7 s were transiently treated with the siRNAs and at 24 hours, assessment of their ability to reduce BCL2 was undertaken. A ß-tubulin loading control is also shown. Results are indicative of two independent experiments. **(f) **MCF-7 cells treated with four independent *BCL2 *siRNAs were grown for 24 hours with 0, 0.1, or 1 mM NaBu as indicated. TUNEL assays were analyzed by flow cytometry to quantify the number of apoptotic cells. Standard deviation is represented by error bars.

**Figure 5:**
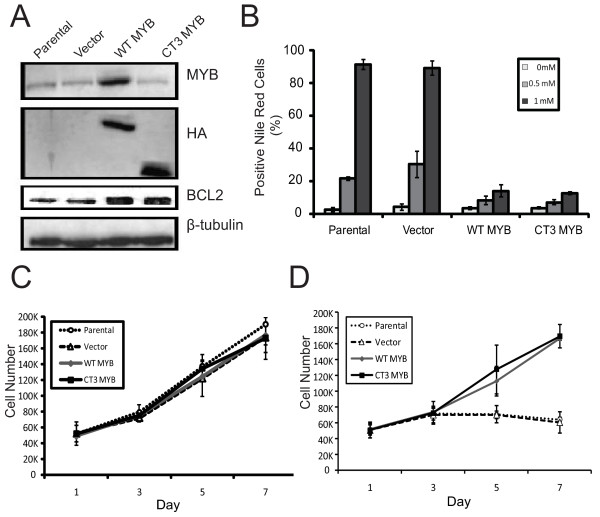
***MYB *overexpression in MCF-7 cells prevents differentiation-inducing agent-induced differentiation and growth arrest**. **(a) **MCF-7 cells were stably transduced with empty pMYS-IRES-GFP vector, or vectors encoding HA-tagged WT-or truncated, activated CT3-MYB. Western blotting with anti-MYB Ab and anti-HA Ab confirmed the expression of exogenous, HA-tagged MYB proteins. Note that the anti-MYB antibody used does not detect CT3-MYB. The Western blot was also probed for BCL2 expression (see text); a ß-tubulin loading control is also shown. **(b) **MYB overexpression blocks lipid accumulation associated with differentiation. Cells were treated with 0.5 mM or 1 mM sodium butyrate (NaBu) for 72 hours, stained with Nile Red and analyzed by flow cytometry. **(c) **Proliferation of MCF-7 cells overexpressing WT or CT3-MYB. MCF-7 cells overexpressing the WT or CT3-MYB, and controls, were grown in normal medium over seven days. **(d) **Proliferation of MCF-7 cells overexpressing the WT or CT3-MYB grown in medium containing 1 mM NaBu over seven days. In (c) and (d) cells were counted on days one, three, five, and seven. Standard deviation is represented by error bars (*n *= 3).

The data above suggest that *MYB *may be protecting mammary epithelial cells against differentiation-associated apoptosis. BCL2 suppresses apoptosis via its interaction with other, pro-apoptotic members of the BCL2 family, and, moreover, several previous studies have shown that MYB regulates *BCL2 *gene expression in hematopoietic cells and in colonic epithelial cells [8,9,31]. We therefore examined *BCL2 *levels by quantitative RT-PCR in MCF-7 cells that had been treated with increasing concentrations of NaBu. As the *MYB *mRNA levels decreased (Figures 1d and 1e), there was a corresponding down-regulation of *BCL2 *mRNA (Figure 4a). We also measured *BCL2 *mRNA levels in the *MYB *knockdown MCF-7 cells, which revealed that induction of *MYB *shRNA resulted in a 65 to 70% reduction compared with controls (Figure 4b). Conversely, overexpression of *MYB *(see below) resulted in increased levels of BCL2 protein (Figure 5a).

Although *BCL2 *is a direct target of *MYB *in T and myeloid cells [32-34] (Zhao *et al*: Defining the MYB Transcriptional Program by Genome-Wide Chromatin Occupancy and Expression Analyses, submitted) this has not been shown in other cell types. To determine whether *MYB *acts directly on the *BCL2 *gene in breast cancer cells, we performed ChIP assays using MCF-7 cells. Potential MBS in *BCL2 *promoters 1 and 2 were identified based on binding sites previously mapped in other cell types [34,35] in conjunction with the established consensus sequence YAACN(G/T) for MYB protein binding [36,37]. Additional sites examined corresponded to those detected by ChIP-Seq in murine myeloid cells (Zhao *et al*, Defining the MYB Transcriptional Program by Genome-Wide Chromatin Occupancy and Expression Analyses, submitted), which are located within intron 2 and downstream of exon 3 (Figure 4c). Following immunoprecipitation of chromatin from MCF-7 cells with anti-MYB antibody, PCR products were detected with the primers for regions in *BCL2 *containing the above-mentioned potential MBS. Quantitative RT-PCR showed substantial enrichment for all four regions and the positive control MYC, but not primers corresponding to the GAPDH gene or a region 6 kb upstream of *BCL2*, with the anti-Myb antibody (Figure 4d). These results demonstrate that endogenous MYB binds *in situ *to the *BCL2 *gene and, combined with the data of Figures 4a, b and 5a (see also below), imply that MYB directly regulates *BCL2 *expression in mammary carcinoma cells.

### *BCL2 *knockdown sensitizes breast cancer cells to DIA-induced apoptosis

The data presented above are all consistent with the proposal that differentiation-associated apoptosis is due in part to loss of *BCL2 *function. To further investigate this hypothesis, siRNAs targeting *BCL2 *were transiently transfected into MCF-7 cells, which were subsequently treated with NaBu. *BCL2 *knockdown was verified by western blot analysis (Figure 4e). When assayed for apoptosis with TUNEL, *BCL2 *knockdown induced 60 to 70% apoptosis in the presence of a level (1 mM) of NaBu that normally would only induce differentiation, whereas only about 20% of cells were TUNEL positive when treated with *BCL2 *siRNA alone (Figure 4f). Thus *BCL2 *knockdown in MCF-7 cells resulted in similar sensitivity to DIA-induced apoptosis to that seen when *MYB *was knocked down using shRNA (Figure 3).

### Enforced expression of *MYB *suppresses differentiation and apoptosis of MECs

To further examine the role of *MYB *in the differentiation of MECs, MCF-7 cells were stably transduced with retroviral vectors expressing either wild type (WT) MYB, a truncated, activated form of MYB (CT3) [38], or the empty pMYs-IRES-GFP vector. Overexpression of MYB was verified by western blot analysis (Figure 5a). The cells were then treated for 72 hours with NaBu. Although MYB overexpression had no effect on proliferation of untreated cells (Figure 5c), Figure 5d shows that overexpression of WT or CT3-MYB allowed the cells to continue proliferating, and prevented differentiation in the presence of NaBu (Figure 5b). As expected, the parental and vector control cells ceased proliferating and underwent differentiation as quantitated by Nile Red staining. These data indicate that overexpression of MYB is capable of preventing induced differentiation of MCF-7 cells.

**Figure 6:**
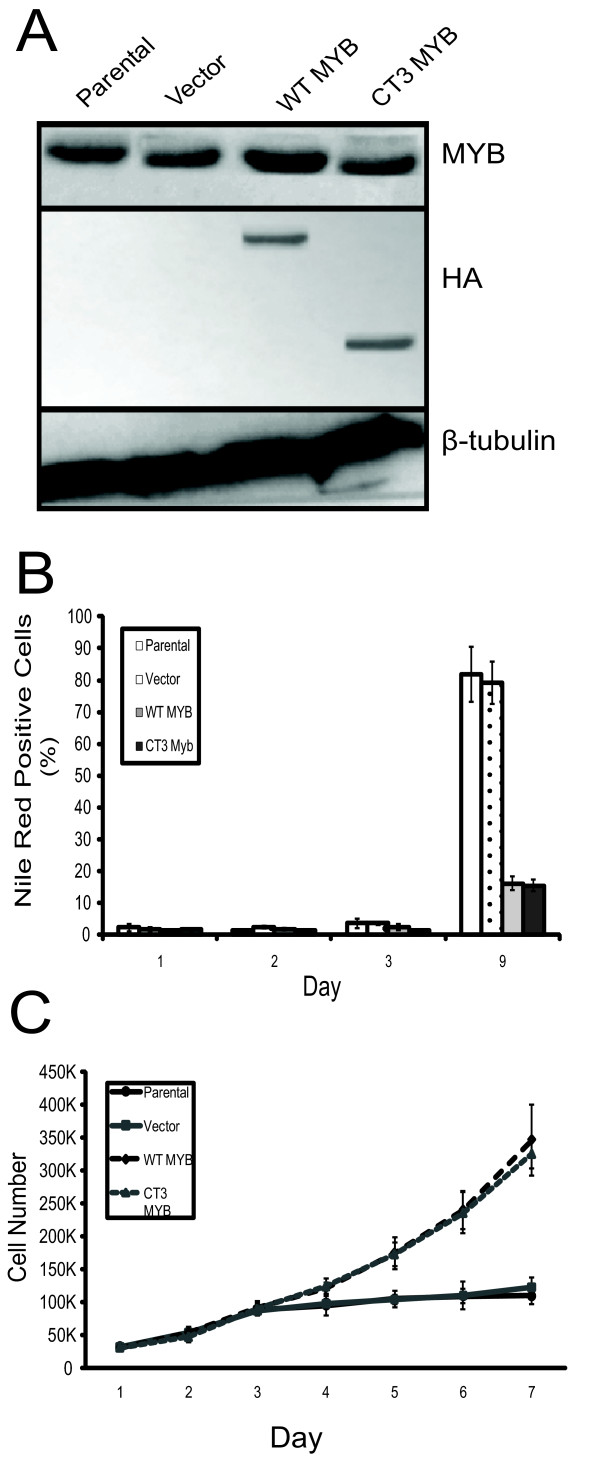
***MYB *overexpression in HC11 cells prevents differentiation and growth arrest**. **(a) **HC11 cells were stably transduced with empty vector, WT-MYB, or CT3-MYB, as in Figure 5. Western blot with anti-MYB Ab and anti-HA Ab confirmed the expression of exogenous and HA-tagged MYB proteins. A ß-tubulin loading control is also shown. **(b) **MYB overexpression blocks lipid accumulation associated with differentiation. Cells were stained with Nile Red at the various stages of differentiation shown, and analyzed by flow cytometry. Standard deviation is indicated by error bars. **(c) **HC11 cells overexpressing WT or CT3-MYB continue to proliferate in differentiation-inducing conditions. HC11 cells as indicated were grown in complete media for two days, after which epidermal growth factor (EGF) was removed for 24 hours and subsequent lactogenic hormones were added to the medium each day Standard deviation is indicated by error bars (*n *= 3).

HC11 cells were also stably transduced with the *MYB *retroviruses, and western blot analysis similarly showed that these cells overexpressed WT or CT3-Myb (Figure 6a). When these cells were induced to differentiate with lactogenic hormones, the WT and CT3-Myb overexpressing cells showed markedly reduced staining with Nile Red compared with controls (Figure 6b), and did not form the domes associated with differentiation (data not shown). The enforced expression of Myb also allowed these cells to continue proliferating in the presence of the lactogenic hormones (Figure 6c); interestingly, proliferation was able to continue in the absence of EGF, although the rate of proliferation was less than that seen in its presence (data not shown).

### Overexpression of *MYB *protects mammary carcinoma cell lines from DIA-induced apoptosis

**Figure 7:**
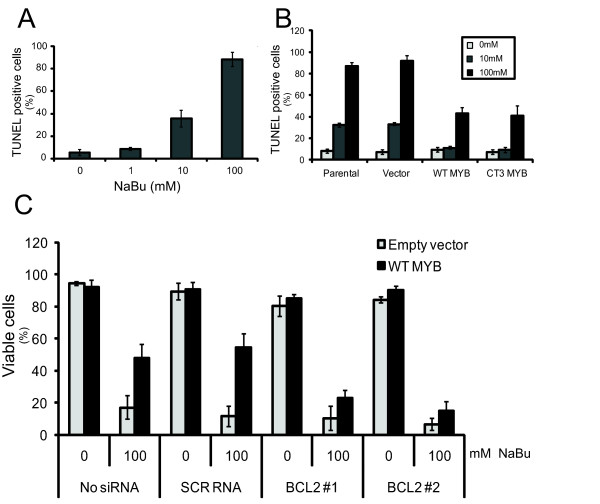
***MYB *overexpression prevents differentiation-inducing agents-induced apoptosis in a *BCL2*-dependent manner**. **(a) **MCF-7 cells were treated with the indicated doses of sodium butyrate (NaBu) for 24 hours before being assayed with TUNEL, which was quantified by flow cytometry. **(b) **MCF-7 cells stably overexpressing WT-MYB, CT3-MYB, or vector, or control parental, were treated with 10 mM or 100 mM NaBu for 24 hours prior to TUNEL assay. **(c) **MCF-7 cells stably overexpressing WT MYB, or control vector, were transiently transfected with siRNAs targeting *BCL2*. They were then treated with 0 or 100 mM NaBu for 24 hours before being assayed with TUNEL. Standard deviation is represented by error bars (*n *= 6).

As the data above showed that enforced *MYB *expression is able to prevent differentiation of MECs, and the data of Figure 3 showed that knockdown of *MYB *results in DIA-induced apoptosis, we asked whether ectopic/overexpression of *MYB *could also protect breast tumor cells against DIA-induced apoptosis. Although the levels of DIAs used in the experiments described above did not induce a significant degree of apoptosis, other reports indicate that higher levels of some of these compounds can do so [39-41]. First, a dose-response assay of DIAs on MCF-7 cells was carried out to determine the level of NaBu required to induce apoptosis in MCF-7 cells at 24 hours (Figure 7a). The MCF-7 WT-MYB and CT3-MYB overexpressing cells, described above, were then treated with apoptosis-inducing levels of each DIA and assayed with TUNEL. Figure 7b shows that overexpression MYB substantially reduced the proportion of cells undergoing apoptosis at the higher concentrations of NaBu. Similar results were obtained with two other DIAs, VES and TPA (data not shown).

Given that *MYB *overexpression results in an increase in the level of BCL2 (Figure 5a), and protects MCF-7 s from differentiation-induced apoptosis (Figure 7b), we next sought to investigate whether *BCL2 *was required for this activity of *MYB*. MCF-7 cells overexpressing WT MYB, or transduced with the empty vector only, were transiently transfected with two of the siRNAs targeting *BCL2 *used previously (Figure 4). These cells were then treated with 0 or 100 mM NaBu for 24 hours before TUNEL assay for apoptotic cells. Figure 7c shows that the protective effect of *MYB *overexpression on cells treated with high levels of NaBu is almost completely abolished when *BCL2 *expression is suppressed.

### Anti-estrogen and DIA treatments synergize to induce differentiation and apoptosis of breast cancer cells

**Figure 8:**
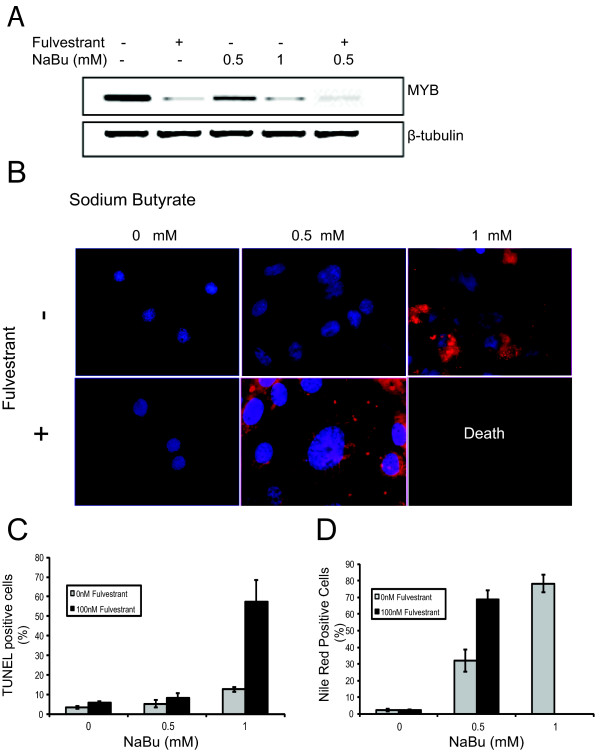
**Fulvestrant synergizes with NaBu to enhance the DIA sensitivity of MCF-7 cells**. **(a) **Western blot analysis of MYB expression in MCF-7 cells treated with fulvestrant or sodium butyrate (NaBu), alone or in combination, as indicated. (The combination of fulvestrant with 1 mM NaBu is not included because of extensive cell loss due to apoptosis. A ß-tubulin loading control is also shown. **(b) **MCF-7 cells grown on glass slides were treated with fulvestrant alone or in combination with 0.5 mM or 1 mM NaBu for 72 hours. The cells were then stained with Nile Red (red) for lipid vesicles and 4',6-diamidino-2-phenylindole (DAPI) (blue) for DNA. **(c) **Apoptosis and **(d) **lipid accumulation were detected by TUNEL assay and Nile Red staining, respectively, and quantitated by flow cytometry standard deviation is represented by error bars (*n *= 3).

As mentioned above, *MYB *expression is directly and positively regulated by estrogen/ER signaling in MCF-7 and other ER positive breast cancer cells [18,19]. We therefore predicted that a pure estrogen antagonist (as distinct to selective estrogen response modifiers such as tamoxifen) might synergize with DIAs in inducing differentiation and apoptosis of such cells. That is, we reasoned that down-modulation of *MYB *resulting from estrogen antagonist treatment would mimic that resulting from shRNA-mediated knockdown. We therefore treated MCF-7 cells with the DIA NaBu, the estrogen antagonist fulvestrant/ICI182780, or both. Reduction of MYB expression by fulvestrant was confirmed by western blotting (Figure 8a). Figure 8b shows that treatment with 0.5 mM NaBu or with fulvestrant alone had little effect, and that as expected 1 mM NaBu induced differentiation but little apoptosis. However, the combination of fulvestrant and 0.5 mM NaBu induced substantial differentiation and importantly, combination with 1 mM NaBu induced extensive apoptosis (Figure 8c). These data are similar to those we obtained by combining shRNA-reduced MYB knockdown with DIA treatment of MCF-7 cells (Figure 3).

## Discussion

### *MYB *regulation of mammary epithelial cell differentiation

The studies reported here have shown that *MYB *has an important role in the control of MEC differentiation and in the resistance of mammary carcinoma cells to apoptosis. As we will discuss further, this role appears to parallel that played by *MYB *in other cell systems where *MYB *expression and function has been extensively characterized - hematopoietic cells and colonic epithelium [4-6,42]. We have confirmed that, like some other cancer cells, mammary carcinoma cells can be induced to differentiate and shown that *MYB *expression decreases during this process. Similarly to the haemopoietic and colonic epithelial cell systems, decreased *MYB *expression on differentiation is not limited to tumor cell lines, as we have shown here for the non-tumorigenic murine MEC line HC11.

A functional role for *MYB *in MEC differentiation is implied by the effects of *MYB *knockdown on breast cancer cell lines. Although a small degree of differentiation was observed with an inducible *MYB *shRNA alone, a more dramatic effect was apparent from the synergy between *MYB *knockdown and (otherwise) marginally-effective concentrations of DIAs, which resulted in essentially complete differentiation. Conversely, enforced *MYB *expression was able to block differentiation of both carcinoma cells and non-tumorigenic HC11 cells, again strikingly paralleling the activities of *MYB *in other cell systems [6,16,43-45].

How *MYB *exerts its effects on differentiation in this system is unknown. One might suspect that the G1 phase growth arrest that accompanies *MYB *knockdown [19] and differentiation [27] [see Additional file 1] in mammary carcinoma cells may be important but is unlikely to be sufficient, because only limited differentiation was observed following *MYB *knockdown alone [see Additional file 2]. In fact, rather less is known about the transcriptional network that regulates differentiation of MECs than in other systems such as hematopoiesis. Thus, our findings suggest that studies to identify *MYB *target genes in MECs would shed light not only on *MYB *function but also on MEC differentiation *per se*. Such targets may be directly bound by MYB (as shown here for *BCL2*) or as in the case of Stat5a-regulated genes, by MYB functioning as a co-activator [20].

### *MYB *and apoptosis of mammary carcinoma cells

When *MYB *knockdown was induced in the presence of DIAs at concentrations that normally induce efficient differentiation but little cell death, apoptosis in over 80% of cells resulted. This was true for all three DIAs (NaBu, VES and TPA) and both cell lines tested, implying that it is a general phenomenon and not the property of one agent or cell line. In any case this observation, taken together with the ability of DIAs at higher concentrations to induce apoptosis by themselves [41,46,47] (Figure 7a), and the ability of *MYB *overexpression to protect against apoptosis, supports the following model. We suggest that DIAs induce pro-apoptotic signals that, at normal differentiation-inducing concentrations, are countered by *MYB *activity, whereas either increased DIA concentrations and hence stronger apoptotic signaling, or reduced *MYB *activity leads to apoptosis. Conversely, increased *MYB *expression overcomes the pro-apoptotic activity of higher DIA concentrations.

Our data and this model raise the question of what the apoptotic signals induced by DIAs are, and how *MYB *protects against them. NaBu has histone deactetylase inhibitor (HDI) activity [48], and indeed our unpublished data show that the HDI suberoylanilide hydroxamic acid (SAHA/Vorinistat) also acts as a DIA in our system. Several mechanisms have been reported for apoptosis induction by HDIs [49], including enhanced expression of pro-apoptotic BH3-only proteins [50]. Furthermore, VES has also been reported to trigger the intrinsic apoptotic pathway, in this case via activation of Bax [51,52]. It is interesting to note that MCF-7 cells are known to be deficient in the apoptotic 'executioner' caspase 3 [53]. However, further work is needed to define the mechanism of DIA-induced apoptosis of breast cancer cells.

*BCL2 *suppresses apoptosis via the intrinsic pathway, and thus regulation of *BCL2 *is a plausible mechanism for the anti-apoptotic function of *MYB *in mammary carcinoma cells. Indeed we have shown here that *BCL2*, a known *MYB *target gene in other cell types, is directly regulated by *MYB *in breast cancer cells, and have identified multiple MBS within the *BCL2 *gene. Moreover we have shown that *BCL2 *is necessary for the ability of *MYB *to protect such cells against DIA-induced apoptosis.

### A common role for *MYB *in multiple tissues and cancers?

As briefly reviewed in the Introduction, and more extensively elsewhere [4-6], *MYB *is essential for the proliferation of multiple cancer and normal cell types, including haemopoietic, colonic and mammary epithelial. Similarly, with the data presented in this report, there is now strong evidence that *MYB *can antagonize differentiation in all three cell systems. *MYB *is also involved in vascular smooth muscle cell proliferation [54] and there are reports of *MYB *down-regulation during differentiation in this system too [55]. Moreover, c-*myb *expression in developing and adult mice has been characterized using *in situ *hybridization and correlated with stage-specific differentiation and mitotic activity [56].

*MYB *can also suppress apoptosis in the hematopoietic and colonic epithelial systems [8,32,57], and, as reported here, in mammary epithelial cells. Data in these systems and those presented here have implicated *BCL2 *as a common effector, although probably not the only one. Whether the other '*MYB *phenotypes' of proliferation and differentiation suppression are mediated by common or specific factors needs to be elucidated. However, it is likely that some tissue-specific factors are involved in effects on differentiation, and indeed this is supported by our recent data in the haemopoietic system (Zhao *et al*: Defining the MYB Transcriptional Program by Genome-Wide Chromatin Occupancy and Expression Analyses, submitted).

### Targeting *MYB *in breast cancer

As discussed above, our data [19] (and that reported here) imply that *MYB *is required in ER positive mammary carcinoma cells for three key 'hallmarks of cancer' [58] - continued proliferation, suppression of differentiation, and resistance to apoptosis. This could potentially make *MYB *an excellent therapeutic target in breast cancer, particularly under conditions where *MYB *activity is limiting for one or more of these processes. The attractiveness of *MYB *as a target in this disease is reinforced by the fact that 60 to 70% of all human breast tumors express *MYB *[17,19] (see also [6,19,59]).

Treating tumors by inducing cancer cell differentiation has been discussed for some time; although an attractive concept, it has rarely proved to be an effective approach by itself. However, the combination of DIAs and other agents can be highly effective; for example, the combination of the DIA all-trans retinoic acid with arsenic trioxide is now the accepted treatment modality for patients with acute promyelocytic leukemia [60]. Our studies have identified the combination of *MYB *inhibition with DIA treatment as a potentially effective combination therapy for ER positive breast cancer that not only suppresses proliferation but induces extensive tumor cell death. Further development will require the identification of more appropriate DIAs for clinical use, and of a feasible approach for inhibiting *MYB *activity in breast tumors. The studies shown here together with our unpublished work suggest that VES and HDIs are both good candidate DIAs for clinical use. Importantly, both have been used in patients and, indeed, the HDI SAHA/Vorinistat is approved for treating cutaneous T-cell lymphoma.

Our observation that fulvestrant, which is in clinical use for breast cancer treatment, appears to substitute in this regard for *MYB *knockdown suggests that the combination of this agent with clinically acceptable DIAs might be one approach to bring this approach to clinical application. This is in apparent contradiction with the data from De los Santos *et al. *[61], which showed that 2.5 mM NaBu induced about 60% of MCF-7 cells to undergo apoptosis, and that there was no synergistic effect of adding fulvestrant. It must be pointed out that the experiments of De los Santos *et al. *were carried out in under estrogen-free conditions. It is likely that the addition of an anti-estrogenic compound would not synergize with a histone deactetylase inhibitor in an already estrogen-free environment. This is further strengthened by the studies by Chopin *et al *[62], which show less than 20% of MCF-7 s cells are apoptotic with 2.5 mM NaBu at 48 hours when in complete (i.e. not estrogen-free) medium. It seems, therefore, that there is clear evidence for a potential chemotherapeutic effect of combining a suitable anti-estrogen and a DIA.

Although we have discussed a number of approaches to targeting *MYB *itself in breast cancer [59], it may instead be possible to target specifically the anti-apoptotic effectors of *MYB *to induce tumor cell killing by DIAs. Our data show that *BCL2 *is a relevant *MYB *target in this regard, raising the possibility of using recently-developed inhibitors of BCL2 [63], such as ABT-737 [64] and its more bioavailable analogue ABT-263 [65], in combination with DIAs. Interestingly, such a combination has recently shown efficacy in a mouse lymphoma model [66]. Further laboratory and animal model studies to assess the effectiveness and potential toxicities of these approaches *in vitro *and *in vivo *are clearly warranted.

## Conclusions

This study has shown that *MYB *knockdown sensitizes breast cancer cells to induced differentiation and apoptosis. Conversely, ectopic *MYB *expression blocks induced growth arrest and differentiation of BC cells. Furthermore, ectopic *MYB *expression blocks apoptosis of breast cancer cells by directly upregulating *BCL2*. These data highlight the potential of combining differentiation inducers and *MYB *inhibition to lead to new breast cancer therapies.

## Abbreviations

ChIP: chromatin immunoprecipitation; DIAs: differentiation-inducing agents; DAPI: 4',6-diamidino-2-phenylindole; DMEM: Dulbecco's Modified Eagle's Medium; Dox: doxycycline; EGF: epidermal growth factor; ER: estrogen receptor; FBS: fetal bovine serum; HDI: histone deactetylase inhibitor; MBS: Myb binding sites; MEC: mammary epithelial cell; NaBu: sodium butyrate; PBS: phosphate-buffered saline; RT-PCR: reverse-transcription polymerase chain reaction; SAHA: suberoylanilide hydroxamic acid; TPA: 12-O-tetradecanoylphorbol-13-acetate; TUNEL: Terminal deoxynucleotidyl transferase (TdT)-mediated dUTP nick end labeling; RPMI: Roswell Park Memorial Institute medium; VES: vitamin E succinate; WT: wild type.

## Competing interests

The authors declare that they have no competing interests.

## Authors' contributions

YD performed laboratory experiment and data analyses. TJG performed data analyses. YD, RGR and TJG initiated and designed the study and were involved in writing the manuscript. All authors have read and approved the final manuscript.

## Supplementary Material

Additional file 1 **Differentiation induction of MCF-7 cells treated with the DIAs VES or TPA**. MCF-7 cells were grown on glass cover slips and treated with increasing amounts of **(a) **vitamin E succinate (VES) or **(b) **12-O-tetradecanoylphorbol-13-acetate (TPA), as indicated, for 72 hours. Fluorescence microscopy (×63) was used to detect. differentiation as assessed by Nile Red staining for lipid vesicles (red). Nuclear DNA was stained with DAPI (blue). **(c to e) **Cell cycle analysis of MCF-7 cells treated with three differentiation-inducing agents (as indicated) at different doses (also as indicated) for 72 hours. Cells were fixed, stained with propidium iodide and analyzed by flow cytometry. The percentage of cells in each cell cycle phase was determined using ModFit. Click here for file

Additional file 2 **The effect of *MYB *knockdown on differentiation in MCF-7 cells**. **(a)**. MCF-7 cells stably transduced with a doxycycline (Dox)-inducible lentiviral siRNA, were grown with or without Dox for 72 hours. The cells were then stained for lipid droplet (Nile Red - Red) or nuclear DNA (DAPI - blue), and evaluated by fluorescent microscopy (×63) or **(b) **Flow cytometry; data for control cell lines are also shown. **(c) **Quantitative PCR analysis of ß-casein mRNA levels following Dox treatment. Data are normalised against untreated cells. Click here for file

Additional file 3 **The effect of *MYB *knockdown on differentiation in ZR-75-1 cells**. **(a) **ZR-75-1 cells stably transduced with a doxycycline (Dox)-inducible lentiviral siRNA, were grown with or without Dox for 72 hours. The cells were then stained for lipid droplet (Nile Red - Red) or nuclear DNA (DAPI - blue), and evaluated by fluorescent microscopy (×63) or **(b) **Flow cytometry; data for control cell lines are also shown. **(c) **Quantitative PCR analysis of ß-casein mRNA levels following Dox treatment. Data are normalised against untreated cells. Click here for file

Additional file 4 **MYB knockdown sensitises ZR-75-1 cells to the differentiation-inducing agent NaBu**. **(a) **ZR-75-1 cells with a doxycycline (Dox)-inducible shRNA targeting *MYB *[19] were grown on a glass coverslip, treated with or without 5 μg/mL Dox for 24 hours before exposure to the indicated concentrations of sodium butyrate (NaBu) for three days. They were then stained with Nile Red (red) for lipid vesicles and DAPI (blue) for DNA detection. **(b) **Flow cytometric analysis of TUNEL staining for apoptotic cells. ZR-75-1 cells stably transfected with inducible shRNA treated for 24 hours with or without Dox, were grown in the presence or absence of 1 mM NaBu for a further 24 hours, and were then assayed for apoptosis using TUNEL. Standard deviation is represented by error bars. Click here for file
